# Production of HlyA and ClyA haemolysins among quinolone-resistant *Escherichia coli* isolated from clinical samples

**DOI:** 10.1186/2193-1801-2-71

**Published:** 2013-02-27

**Authors:** Alicia Márquez-López, Belén Ruiz del Castillo, María Eliecer Cano, Cristina Rodríguez-Mirones, Jesús Oteo, David Sáez, Luis Martínez-Martínez

**Affiliations:** 1 Service of Microbiology, University Hospital Marqués de Valdecilla-IFIMAV, Santander, Spain; 2 Departament of Molecular Biology, University of Cantabria, Santander, Spain; 3 Antibiotic Laboratory, Bacteriology Service, Centro Nacional de Microbiología, Instituto de Salud Carlos III, Madrid, Spain

**Keywords:** Haemolysis, *Escherichia coli*, Quinolone resistance, *hlyA*, *clyA*

## Abstract

Most *Escherichia coli* resistant to quinolones are not haemolytic. The objective of this study was to determine the phylogroup, clonal relationship, mechanism of quinolone resistance and virulence factors in 70 haemolytic *E. coli* resistant to nalidixic acid. Sixty-six isolates contained the *hlyA* gene, belonged to phylogroup B2, and 61 of them presented low-level resistance to fluoroquinolones. Four isolates presented high-level resistance to fluoroquinolones, contained the *clyA* gene and were included in phylogroup D. One single isolate (phylogroup D, with low level resistance to fluoroquinolones) contained both cytotoxins.

**Electronic supplementary material:**

The online version of this article (doi:10.1186/2193-1801-2-71) contains supplementary material, which is available to authorized users.

*Escherichia coli* strains causing extraintestinal infections (ExPEC) express different virulence factors (VF), including α-haemolysin (HlyA) and cytolysin A (ClyA, SheA or HlyE), both of which can induce osmotic lysis of erythrocytes (Kerényi et al. 2005). However, quinolone-resistant clinical isolates of *E. coli* ExPEC are frequently non-haemolytic (Horcajada et al. 2005; Martínez-Martínez et al. 1999). This may be related to the observation that strains of phylogroup B2 and D usually contain more VF (including haemolysins) than those of phylogroups A and B1 (Clermont et al. 2000; Houdouin et al. 2007). On the other hand, isolates of groups A and B1 are more frequently resistant to quinolones than those of phylogroup B2 and D (with the notable exception of some particular multiresistant clones, such as the O25:H4-B2-ST131 clone) (Houdouin et al. 2007; Takahashi et al. 2009). Thus, a detailed analysis of clinical isolates presenting the unusual association of quinolone resistance and haemolysis production may be helpful to understand the relationship between virulence and resistance in *E. coli*.

From November 2002 to March 2010, 33042 *Escherichia coli* were isolated from clinical samples in our laboratory (University Hospital Marqués de Valdecilla, Santander, Spain). Seventy (0.2% of all isolates) were haemolytic and resistant to nalidixic acid. Organisms were cultured from urine (63 isolates), blood (2), soft tissue abscess (1), abdominal abscess (1), wound (1), tracheal aspirate (1) or skin ulcer (1). Bacteria were identified with the Microscan WalkAway 96 system (Dade Behring, CA, USA). MICs of nalidixic acid (Sigma-Aldrich, Madrid, Spain), ciprofloxacin (Sigma-Aldrich), norfloxacin (Sigma-Aldrich) and levofloxacin (Aventis Pharma, Madrid, Spain) were determined by broth microdilution, according to CLSI guidelines (Clinical and Laboratory Standards Institute 2011). Haemolysin production was assessed in sheep blood agar (Oxoid, Madrid, Spain). An organism was considered haemolytic when a clear halo was observed around isolated colonies after overnight incubation at 37°C. The phylogroup was determined by multiplex PCR (Clermont et al. 2000). Clonal relationship was evaluated by repetitive extragenic palindromic PCR (REP-PCR); isolates were considered unrelated if more than two bands of difference were observed. Additionally, 27 isolates representative of the different patterns obtained by REP-PCR (1–4 isolates per REP-PCR pattern), were typed by Pulsed-Field Gel Electrophoresis (PFGE) and analyzed by Multilocus Sequence Typing (MLST) according to the protocol specified at the *E. coli* MLST website (http://mlst.ucc.ie/mlst/dbs/Ecoli). This resulted in the recognition of 13 REP-PCR pattterns, 14 PFGE patterns and 11 sequence types (ST), which combined allowed to define 15 distinct organisms. The quinolone resistance-determining regions (QRDRs) of *gyrA* and *parC* were amplified (Vila et al. 1996a; Vila et al. 1996b) and sequenced in the Molecular Genetics Unit of the HUMV. The presence of genes coding for five major horizontally transmissible quinolone resistance determinants (*qnrA*, *qnrB*, *qnrS*, *qepA*, and *aac-(6′)-Ib-cr*) was investigated by multiplex PCR (Cano et al. 2009). The presence of the *hlyA* and *clyA* genes was evaluated by PCR (Kerényi et al. 2005). Additionally, 19 virulence-associated genes (*traT, iutA*, *bmaE*, *iroN*, *sfaS*, *afa/dra, ibeA, fyuA*, *fimH*, *Pai, K1*, *K5, KpsMTII*, *KpsMTIII, cnf1*, *focG*, *gafD*, *papC*, *sat*) were analyzed in the previously indicated 15 isolates (Johnson & Stell 2000; Bonacorsi et al. 2003). The VF score of each isolate was calculated as the number of VF genes for which the isolate tested positive (Bert et al. 2008).

The distribution of MICs of the four evaluated quinolones is presented in Additional file 1. According to the CLSI breakpoints, 65 (92.8%), 64 (91.4%) and 66 (94.3%) out of the 70 isolates were susceptible to ciprofloxacin, norfloxacin and levofloxacin, respectively. When the breakpoints defined by EUCAST were considered, the percentages of susceptibility to ciprofloxacin, norfloxacin and levofloxacin were 88.6%, 54.3% and 91.4%, respectively. All isolates contained one or two mutations in *gyrA* alone or (in six cases) associated to a mutation in *parC* (Table 1). The most common change in GyrA was serine to leucine in position 83. Plasmid-mediated quinolone resistance determinants were not detected. Interestingly, although all our haemolytic isolates contained one or more DNA-gyrase mutations, most of them are defined as susceptible to fluoroquinolones according to the CLSI breakpoints (Clinical and Laboratory Standards Institute 2011), or even to the more restrictive breakpoints defined by EUCAST (European Committee on Antimicrobial Susceptibility Testing 2012).

**Table 1:** **Phylogroup (PhG), clonal relationship defined by REP-PCR patterns (REP), Pulsed-Field Gel Electrophoresis (PFGE), and Multilocus Sequence Typing (MLST), MIC of quinolones, mutations in topoisomerase genes, and presence of haemolysins and virulence-associated genes in a representative set of isolates**

				MLST		MIC (μg/ml)				Mutations		Haemolysins		Virulence-associated genes								
**Isolate**	**PhG**	**REP (n)**	**PFGE**	**ST**	**ST cplx**	**NAL**	**NOR**	**CIP**	**LEV**	*gyrA*	*parC*	*hlyA*	*clyA*	*traT*	*iroN*	*ibeA*	*fyuA*	*cnf1*	PAI	*papC*	*K5*	*KpsMTII*
Hly-557	D	A (4)	1B	350	350	>512	>16	16	8	S83L D87N	S80I	-	+	-	-	+	-	-	-	-	-	-
Hly-1872	D		2	57		>512	>16	8	4	S83L D87N	S80I	-	+	+	-	+	-	-	-	-	-	-
Hly-263	D	B (1)	3	405	405	32	0.5	0.125	0.125	D87G	/	+	+	+	-	+	+	+	+	+	+	+
Hly-520	B2	C (16)	4A	12	12	256	0.5	0.25	0.25	S83L	/	+	-	+	+	-	+	+	+	+	+	+
Hly-6129	B2		5			256	1	0.25	0.25	S83L	/	+	-	-	+	-	+	+	+	+	-	-
Hly-2304	B2	D (1)	6	599	12	>512	>16	1	1	S83L	/	+	-	+	-	-	+	+	-	+	-	-
Hly-4105	B2	E (19)	7	372	None	128	0.5	0.25	1	S83L	/	+	-	-	+	+	+	+	+	+	-	-
Hly-2425	B2	F (7)	8A	372	None	128	1	0.25	0.25	S83L	/	+	-	+	+	+	+	+	+	+	+	+
Hly-4530	B2	G (10)	9	73	73	128	0.5	0.25	0.25	S83L	/	+	-	+	+	-	+	+	+	+	+	+
Hly-3308	B2	H (19)	10C	73	73	64	0.5	0.125	0.125	D87Y	/	+	-	+	+	-	+	+	+	-	+	+
Hly-5126	B2	I (2)	11	127	None	64	1	0.25	0.5	D82N	/	+	-	+	+	-	+	+	+	+	+	+
Hly-3606	B2	J (1)	12	127	None	64	0.5	0.25	0.25	S83L	/	+	-	+	+	-	+	+	+	+	+	+
Hly-2605	B2	K (6)	13C	131	None	256	1	0.25	0.5	S83L	/	+	-	+	+	+	+	+	+	+	+	+
Hly-3438	B2	L (1)	13D			128	0.5	0.25	0.25	S83L	/	+	-	+	+	+	+	+	+	+	+	+
Hly-1248	B2	M (1)	14	537	14	>512	4	1	0.5	S83L	S80R	+	-	+	+	+	-	+	-	+	+	+

ST = Sequence Typing, ST cplx = Sequence Typing complexes, NAL = nalidixic acid, NOR = norfloxacin, CIP = ciprofloxacin, LEV = levoflofacin, S = Serine, L = Leucine, D = Aspartic acid, N = Asparagine, I = isoleucine, G = glycine, Y = tyrosine and R = arginine.

Sixty-six (94.3%) and five (7.2%) isolates corresponded to phylogroups B2 and D. Most isolates of phylogroup B2 belonged to ST73, ST12, ST372 or ST131, while the majority of the isolates of the group D belonged to ST350 (Table 1). Molecular typing has shown a certain degree of clonal diversity among the isolates we have evaluated, with some clones containing single isolates and some other including multiple isolates. According to these data, a qualitative analysis indicates that our isolates do not represent the dissemination of only one single or just a few clones.

Sixty-five (92.9%) isolates contained the *hlyA* gene, four (5.7%) isolates contained the *clyA* gene and one (1.4%) single isolate contained both *hlyA* and *clyA*. In a previous study by Kerényi et al. on 540 extraintestinal *E. coli* strains, the *clyA* gene was identified in 241 isolates and *hlyA* in 198 isolates (Kerényi et al. 2005); these authors did not find an isolate simultaneously containing both haemolysin genes and suggested their possible incompatibility, which is in contrast with our observation of an isolate with the two haemolysin genes. MICs of ciprofloxacin for 61 out of 65 (93.8%) isolates with only *hlyA* were ≤ 0.5 μg/ml while MICs of this agent against the four isolates containing only *clyA* were ≥ 8 μg/ml. The MICs of the single isolate with both haemolysins was 0.125 μg/ml. It is difficult to determine if the presence of *clyA* alone is more common in isolates with high level fluoroquinolone resistance, as we have found this association in just a small number of isolates (all of them of ST 350). It would be possible that this relationship is related to the phylogenetic background of the isolates, as *hlyA* was linked to phylogroup B2, while *clyA* was linked to phylogroup D. Additional studies are in progress to evaluate this observation in a large collection of *E. coli* consecutively isolated from bacteremic episodes. This analysis would also provide more data on the presence of *hlyA* in strains with just low-level resistance to fluoroquinolones.

The VF score was slightly higher for isolates of phylogroup B2 (10.9) than for those of phylogroup D (7.6). In the 15 isolates studied in detail, the most frequent virulence-associated genes (Table 1) were *fimH* (100%), *cnf1* (87%), *traT* (80%), *fyuA* (80%) and *papC* (80%). None of the isolates presented *kpsMTIII*. Other genes such as *PAI* (73%), *iroN* (73%), *K5* (67%), *kpsMTIII* (67%), *ibeA* (53%), *iutA* (46.7%), *sfaS* (33.3%), *focG* (26.6%), a*fa/dra* (20%), *sat* (13.3%), *bmaE* (6.6%), *K1* (6.6%) and *gafD* (6.6%) were less frequently detected. Isolates with *clyA* also contained more frequently *iutA* and *ibeA*. Previous studies have shown that the *fimH* and *traT* genes are more frequently found in *E. coli* isolates (irrespective of their susceptibility to quinolones) than other virulence genes such *cnf1* or *hlyA* (Takahashi et al. 2009; Cooke et al. 2010). The prevalence of *cnf1* in this study, which is higher than that previously described in isolates causing urinary and skin infections could be explained by the association of *cnf1* and *hlyA* in a pathogenicity island, as previously reported (Johnson & Stell 2000; Petkovsek et al. 2009; Schmidt & Hensel 2004).

## Electronic supplementary material

Additional file 1: Table 1: Distribution of absolute (and cumulative) MICs (μg/ml) of four quinolones against 70 haemolytic *E. coli* isolates. Values corresponding to resistance and intermediate susceptibility (according to CLSI criteria) are shadowed in dark and pale grey, respectively. (PDF 25 KB)
