# Supplementary material for: Production of HlyA and ClyA haemolysins among quinolone-resistant Escherichia coli isolated from clinical samples
Source: Springerplus. 2013 Feb 27;2:71. doi: 10.1186/2193-1801-2-71 (PMC3607716; doi:10.1186/2193-1801-2-71)
Supplement: Supplementary file 1 — Additional file 1: Table 1: Distribution of absolute (and cumulative) MICs (μg/ml) of four quinolones against 70 haemolytic E. coli isolates. Values corresponding to resistance and intermediate susceptibility (according to CLSI criteria) are shadowed in dark and pale grey, respectively. (PDF 25 KB) [file 40064_2013_163_MOESM1_ESM.pdf]

Table 1. Distribution of absolute (and cumulative) MICs (µg/ml) of four quinolones against 70 haemolytic *E. coli* isolates. Values corresponding to resistance and intermediate susceptibility (according to CLSI criteria) are shadowed in dark and pale grey, respectively.

| **Quinolone** | **Phylogroup (n)** | **MIC (µg/ml)** | | | | | | | | | | |
|---|---|---|---|---|---|---|---|---|---|---|---|---|
| **Nalidixic acid** | | **<=1** | **2** | **4** | **8** | **16** | **32** | **64** | **128** | **256** | **512** | **>512** |
| | **B2 (65)** | | | | | | | 13 | 18 (31) | 23 (54) | 8 (62) | 3 (65) |
| | **D (5)** | | | | | | 1 | | | | | 4 (5) |
| **Ciprofloxacin** | | **<= 0.03** | **0.06** | **0.125** | **0.25** | **0.5** | **1** | **2** | **4** | **8** | **16** | **>16** |
| | **B2 (65)** | | 3 | 16 (19) | 29 (48) | 13 (61) | 4 (65) | | | | | |
| | **D (5)** | | | 1 | | | | | | 3 (4) | 1(5) | |
| **Levofloxacin** | **B2 (65)** | | 1 | 16 (17) | 30 (47) | 14 (61) | 2 (63) | 2 (65) | | | | |
| | **D (5)** | | | 1 | | | | | 3 (4) | 1 (5) | | |
| **Norfloxacin** | **B2 (65)** | | | | 4 | 33 (37) | 21 (58) | 4 (62) | 1 (63) | | 1 (64) | 1 (65) |
| | **D (5)** | | | | | 1 | | | | | | 4 (5) |
